# Cooperative Sorption on Heterogeneous Surfaces

**DOI:** 10.1021/acs.langmuir.2c01750

**Published:** 2022-10-18

**Authors:** Olivia P. L. Dalby, Steven Abbott, Nobuyuki Matubayasi, Seishi Shimizu

**Affiliations:** † York Structural Biology Laboratory, Department of Chemistry, University of York, Heslington, York YO10 5DD, United Kingdom; ‡ Steven Abbott TCNF Limited, 7 Elsmere Road, Ipswich, Suffolk IP1 3SZ, United Kingdom; § School of Mechanical Engineering, University of Leeds, LeedsLS2 9JT, United Kingdom; ∥ Division of Chemical Engineering, Graduate School of Engineering Science, Osaka University, Toyonaka, Osaka560-8531, Japan

## Abstract

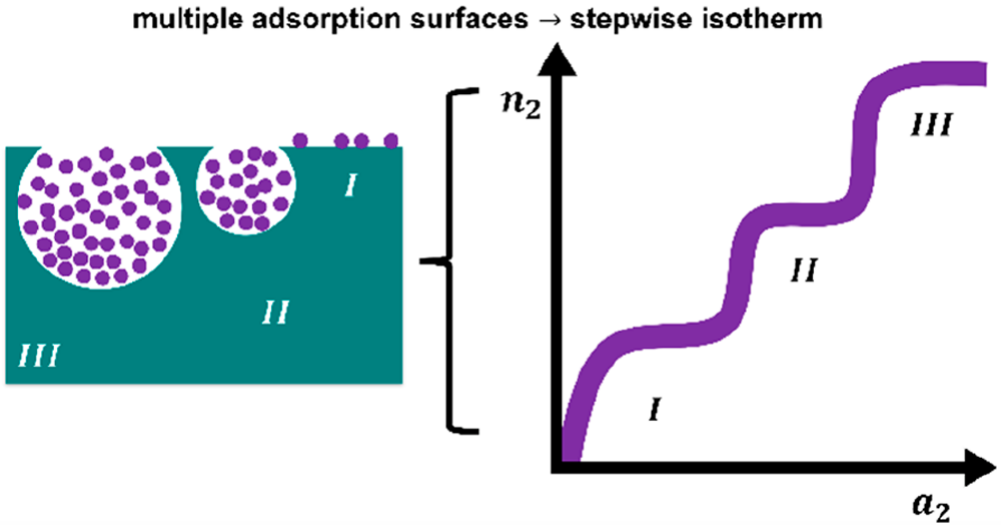

Heterogeneous adsorbents, those composed of multiple surface and pore types, can result in stepwise isotherms that have been difficult to model. The complexity of these systems has often led to appealing to empirical equations without physical insights, unrealistic assumptions with many parameters, or applicability limited to a particular class of isotherms. Here, we present a statistical thermodynamic approach to model stepwise isotherms, those consisting of either an initial rise followed by a sigmoid or multiple sigmoidal steps, founded on the rigorous statistical thermodynamic theory of sorption. Our only postulates are (i) the finite ranged nature of the interface and (ii) the existence of several different types of microscopic interfacial subsystems that act independently in sorption. These two postulates have led to the superposition scheme of simple surface (i.e., Langmuir type) and cooperative isotherms. Our approach has successfully modeled the adsorption on micro–mesoporous carbons, gate-opening adsorbents, and hydrogen-bonded organic frameworks. In contrast to the previous models that start with *a priori* assumptions on sorption mechanisms, the advantages of our approach are that it can be applied universally under the above two postulates and that all of the fitting parameters can be interpreted with statistical thermodynamics, leading to clear insights on sorption mechanisms.

## Introduction

Sorption isotherms are ubiquitous across a range of disciplines, e.g., food moisture content at various humidities,^1−3^ water uptake into hardened cement paste,^4,5^ or drug delivery systems^6^ and pharmaceutical excipients.^7^ However, explaining and modeling the functional shape of an isotherm from the underlying interactions on a molecular scale is a challenging task, in view of the diverse functional shapes [the six International Union of Pure and Applied Chemistry (IUPAC) isotherm types^8,9^]. This task was made even more difficult by the co-existence of multiple isotherm models (i.e., more than 80 isotherm models were already proposed by 1981^10^) of different scopes and premises (physical, empirical, and semi-empirical).^11,12^ Our goal, instead, is to present a unified, systematic approach, applicable to all types of sorption isotherms, based on statistical thermodynamics.^13−16^ Recently, we have proposed a statistical thermodynamic approach to model the sorption isotherms on homogeneous surfaces, such as the IUPAC Types I and II [for which the Langmuir, Brunauer–Emmett–Teller (BET), and Guggenheim–Anderson–de Boer (GAB) models have been used]^14^ as well as Type V (which exhibits capillary condensation with a weak adsorbent–adsorbate interaction).^16^ In this paper, we extend our statistical thermodynamic approach to more complex isotherms that involve multiple sorption steps, such as Type IV (traditionally understood as the monolayer–multilayer transition followed by capillary condensation) and Type VI (and Type VI-like) multiple stepwise sorption isotherms, both resulting from heterogeneous adsorbents.^8,9^

We will summarize below why the isotherms that involve multiple sorption steps have been particularly difficult to model and how the difficulties can be overcome by the statistical thermodynamic theory.

### Type IV Isotherms

#### Sorbate–Sorbent Interaction at Low Activity

Among the isotherms that exhibit a single step, a Type IV isotherm is distinguished from a Type V isotherm by a stronger sorbate–sorbent interaction that leads to the increase of sorption at low sorbate activity.^8,9^ This has traditionally been referred to as the “initial monolayer–multilayer adsorption on the mesopore walls”.^8,9^ Therefore, to model a Type IV isotherm, both sorbate–sorbent interaction at the low-activity region and capillary condensation at higher activity must be captured. The existing model-based approaches to Type IV isotherms can be classified into the following categories.

#### Empirical and Semi-empirical Models

With recognition that the Type IV isotherm consists of two separate processes (i.e., monolayer–multilayer and capillary condensation), a superposition of Henry’s law contribution and the Sips isotherm,^17^ Langmuir and Sips isotherms,^17,18^ or multiple Dubinin–Astakhov isotherms^19^ have been adopted. Here, superposing isotherm models linearly has been carried out on a pragmatic basis. Moreover, both the Sips and Dubinin–Astakhov models are empirical mathematical relationships that relate sorbate activity or adsorption potential to an isotherm.^15^ Yet, neither the Sips nor Dubinin–Astakhov model satisfies Henry’s law at a low sorbate activity limit.^15^ Consequently, even though the empirical models are beneficial in their ability to fit experimental data, gaining mechanistic insight into sorption is beyond their premises.^13−16^

#### Physical Models

Despite the multitude of isotherm models proposed, many have failed to fit Type IV isotherms, as reviewed in detail.^20−23^ Relatively few models have proven to be successful. Do and Do assumed the partial detachment of adsorbed water clusters, in which one part binds onto an active site, while another engages in pore filling.^24,25^ Additionally, Buttersack, generalizing the Klotz isotherm from protein–ligand binding,^26,27^ derived an equation capable of fitting Type IV and V isotherms,^22,23^ equivalent in form to the ζ isotherm of Ward and Wu.^28^ The basic assumption of the Klotz-derived isotherm is a successive binding of sorbates (up to a finite number) onto the binding sites.^22,23^ How stepwise binding constants depend upon the sorbate cluster size can be determined from an energetic model of capillary condensation.^22,23^ However, despite the successes of these models in fitting experimental data, each model introduces a different set of assumptions on adsorption mechanisms, as summarized above. The co-existence of multiple isotherm models founded upon different assumed mechanisms, with the only measure of success being the goodness of fit,^29^ makes it difficult to identify the underlying adsorption mechanism, just as in the case for Types I and II isotherms.^13−16^

### Multiple Stepwise Isotherms

#### Origin of Multiple Sorption Steps

There are two classes of sorption phenomena leading to stepwise isotherms. The first is the (narrowly defined) Type VI isotherm, which, according to IUPAC, is “representative of layer-by-layer adsorption on a highly uniform nonporous surface. [...] Amongst the best examples of Type VI isotherms are those obtained with argon or krypton at low temperature on graphitized carbon blacks”.^9^ The second is a class of gas adsorption isotherms, often at room temperature, on porous materials, such as micro–mesoporous carbons,^30,31^ metal–organic frameworks,^32^ porous coordination polymers,^33^ and zeolites.^34^ Note that this second class of multiple-step isotherms is outside the original IUPAC definition of Type VI yet exhibits a similar functional shape. Here, the stepwise behavior has been attributed to the filling of different sized pores^30−32^ or surface structural changes caused by adsorption.^33^

#### Models for Stepwise Isotherms

Physical models have been proposed to fit stepwise isotherms. Pera-Titus et al. derived a heterogeneous isotherm thermodynamically, whose model assumptions involve multiple parameters for energy heterogeneity and sorbate/sorbent interaction for sorbate-induced structural changes to zeolites, necessitating a large array of fitting parameters for each region of an isotherm plot.^34^ Ng et al. developed a “universal” model to fit all isotherms based on the hypothesized balance of adsorption and desorption rates on local adsorption sites, which has led to the superposition of Langmuir-type isotherms.^35^ Ben Yahia et al. developed a statistical model specially for Type VI isotherms (i.e., adsorption on highly uniform surfaces at a low temperature), in which the stepwise nature of the isotherm was reproduced by assuming multiple adsorption energy levels^36^ yet has led to different forms of fitting equations for each experimental data set, as pointed out by Ng et al.^35^

### Our Approach

We have seen that multiple isotherm models and approaches co-exist for adsorption on cooperative and stepwise isotherms, in which the assumed sorption mechanism differs from model to model. Capturing surface heterogeneity (i.e., the existence of different surface types, such as micro- and mesopores) that leads to multiple sorption steps (such as “monolayer–multilayer” adsorption and capillary condensation or pore filling of different sizes), which may involve structural changes, has been challenging, as also summarized above. In contrast to the previous model-based approaches, our goal is to establish a universal approach, founded on two postulates of (i) the finite ranged nature of the interface and (ii) the existence of several different types of microscopic interfacial subsystems that act independently at sorption, to model all IUPAC isotherm types. In our recent papers, we have proposed a universal approach to interpret sorption isotherms based on quantifying the underlying sorbate–sorbent and sorbate–sorbate interactions via the fluctuation theory.^13,15,37^ This has led to two general approaches to model isotherms: cluster expansion of the sorbate–sorbate interaction^14^ and the cooperative sorption theory,^16^ thereby covering IUPAC Types I, II, and V. In this paper, we will generalize our cooperative sorption theory^16^ to model heterogeneous and stepwise isotherms. On the basis of statistical thermodynamics, we propose the two universal measures of cooperative sorption: (i) the sorbate cluster number and (ii) the free energy of transferring the cluster from the saturated sorbate vapor to the interface. These measures are determined by well-defined expressions of statistical thermodynamics and can form a basis for assessing intermolecular interactions at adsorption without resorting to ad hoc mechanisms of sorption. The theory described here has been implemented in a freely available open-source versatile app to allow users to test the ideas using their own data sets, loaded into the app as simple .csv files. Not only does the interactive app carry out fitting, but it also demonstrates statistical thermodynamics at work in an interactive manner.

## Theory and Methods

### Fluctuation Sorption Theory

Our goal is to develop a universal approach to model sorption isotherms that have been considered to be challenging., i.e., IUPAC Type IV and stepwise isotherms. Our starting point is the rigorous statistical thermodynamic theory, the fluctuation sorption theory.^13−16^ Here, we briefly outline the theoretical foundation, with the detailed theoretical derivations to be referred to in our recent papers.^13−16^ Our foundation is the generalized Gibbs isotherm valid for any interface regardless of surface geometry and porosity.^13^ This was made possible by formulating the Gibbs dividing surface statistical thermodynamically via the Legendre transform, instead of conventionally based on the concentration profile.^13^ We postulate that the interfacial effect is local; i.e., the deviations from the bulk of the sorbate–interface and sorbate–sorbate distribution functions are confined within the local subsystem, within a finite distance from the interface.^13^ On the basis of this setup, the isotherm is the sorbate activity (*a*_2_, with the species indexes 1 and 2 for sorbent and sorbate, respectively^13−16^) dependence of ⟨*n*_2_⟩ (i.e., the ensemble average of the sorbate number in the local interfacial subsystem, *n*_2_), which obeys the following fundamental relationship:
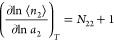
1awhere *N*_22_ is the excess number of sorbates around a probe sorbate molecule, defined as^13^
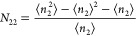
1band the sorbate number fluctuation (⟨*n*_2_^2^⟩ – ⟨*n*_2_⟩^2^ – ⟨*n*_2_⟩) has been used as the measure of the sorbate–sorbate interaction.^13−16^ We are choosing to use the standard thermodynamic nomenclature for consistency with our other papers. In IUPAC nomenclature, *a*_2_ is *p*/*p*_0_ and ⟨*n*_2_⟩ is *n*. Quantifying statistical interactions via fluctuation is common to a well-established approach to solutions, macromolecules, and colloids.^38−41^ Note that our goal is to fit the overall shape of an isotherm based on statistical thermodynamics; hence, our focus is mainly on the functional shape of the isotherm in the finite *a*_2_ region, where ⟨*n*_2_⟩ is the dominant contribution to the surface excess.^37^

### Sorption Polynomial

The key to the statistical thermodynamic approach to modeling isotherms is the sorption polynomial, which is the generalization of the binding polynomial used widely in protein–ligand binding.^42−47^ Although the binding polynomial is founded on the grand canonical partition function, it was interpreted in practice as the successive and stepwise binding of multiple ligands on the binding sites.^42−47^ Such an assumption reflects the reality for protein–ligand binding with well-defined binding sites but not for cooperative sorption via capillary condensation onto porous materials that often contain a very small amount of surface functional groups.^48^ Consequently, the sorption polynomial σ(*T*,*a*_2_) can be derived directly on the basis of the partition function of sorbates in the local interfacial subsystem without involving any assumptions on binding sites or binding constants, as in the following:^16^
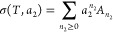
2where *A*_*n*_2__ comes from the partition function of *n*_2_ sorbates in the local subsystem.^16^ The sorption polynomial σ(*T*,*a*_2_) is linked to the isotherm via
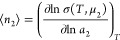
3which is our fundamental relationship for modeling isotherms.^16^

### Modeling Cooperative Isotherms

Here we summarize how cooperative isotherms (namely, the single-step IUPAC Type V without an increase at low sorbate activity) can be modeled with statistical thermodynamics.^16^ First, the amount of sorbate ⟨*n*_2_ ⟩ is an extensive quantity that scales with the quantity of sorbent (this is consistent with the practice of reporting isotherms per unit quantity of sorbent^16^). According to eq 3, the extensive nature of ⟨*n*_2_⟩ is equivalent to extensive ln σ(*T*,*a*_2_), which leads to the multiplicative nature of σ(*T*,*a*_2_) as
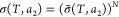
4in terms of σ̃(*T*,*a*_2_), the sorption isotherm of the interfacial unit is introduced, and there are *N* statistically independent interfacial units that constitute the interfacial subsystem.^16^ Second, we postulate the microscopic nature of the interfacial unit, which contains a finite number of sorbates.^16^ Consequently, the sorption isotherm σ̃(*T*,*a*_2_) for the microscopic interfacial unit contains finite terms as
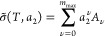
5with *m*_max_ being the maximum order.^16^ Because the subsystem is finite in size, there is an upper limit *m*_max_ to the number of sorbates therein.^16^ Third, postulating the dominance of the contribution from *A*_*m*_*a*_2_^*m*^ (where *m* ≤ *m*_max_) and incorporating *A*_1_*a*_1_ have led to the following isotherm equation:
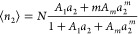
6Equation 6 has successfully modeled Type V isotherms of water sorption isotherms on porous carbons.^16^

### Modeling Stepwise Isotherms

**Figure 1:**
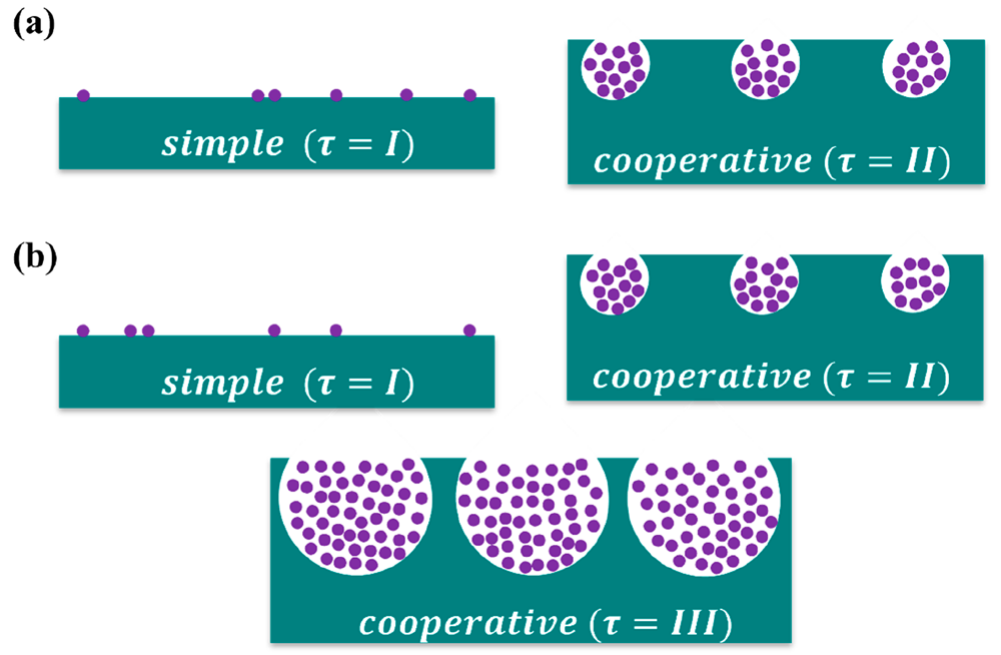
Schematic depiction of the adsorption processes behind the heterogeneous cooperative sorption theory. (a) Adsorption processes for eq 11: a simple surface (*I*) and a cooperative porous component (*II*). (b) Adsorption processes for eq 12: a simple surface (*I*), a cooperative porous component (*II*), and another cooperative component from a different pore size (*III*).

Here, we generalize the cooperative sorption theory (eq 6)^16^ to stepwise isotherms. To do so, we generalize eq 4 by postulating that there are multiple types of microscopic interfacial units (indexed with τ). Consequently, σ(*T*,*a*_2_) is expressed in terms of multiple types of microscopic sorption polynomial σ̃^(τ)^(*T*,*a*_2_) as
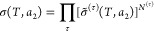
7where *N*^(τ)^ is the number of type τ microscopic interfacial units within the interface. Because σ̃^(τ)^(*T*,*a*_2_) is microscopic, there is a maximum order, ν_max_^(τ)^, within the sorption polynomial as
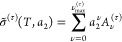
8Using eq 3, the sorption isotherm can be expressed as
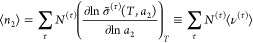
9with the functional shape of the microscopic ⟨ν^(τ)^⟩ given as
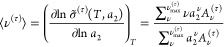
10Equation 9 serves as the foundation for adding isotherm contributions to model more complex isotherms, which has been invoked empirically before. Our basic postulate is still the existence of statistically independent interfacial units of the microscopic scale (eq 4)^16^ that have been generalized here (eqs 9 and 10) to heterogeneous interfaces through the introduction of multiple types of microscopic interfacial units that are statistically independent. This approach, intuitively speaking (Figure 1), is equivalent to assuming that the adsorptions onto different pores and surface adsorption sites (i.e., the interfacial units) are statistically independent.

### Isotherm Functions for Fitting

Modeling Type IV isotherms requires two contributions. The first, indexed as τ = *I*, is simple surface adsorption, a Langmuir-type isotherm, which can be obtained by setting ν_max_^(*I*)^ = 1 in eq 10. For the second, indexed as τ = *II*, we adopt a cooperative isotherm (eq 6) with *A*_1_ = 0 in practice (see below), because Henry’s law is satisfied already by the first contribution. Thus, we obtain
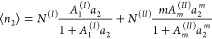
11Modeling multiple stepwise isotherms requires another cooperative term, indexed as τ = *III*, in addition to eq 11, which leads to the following form:
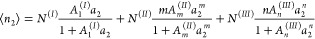
12See Figure 1 for a schematic representation of the processes behind eqs 11 and 12. All of the parameters in eqs 11 and 12 have a clear physical meaning: ν^(τ)^ (= *m* or *n* in eqs 11 and 12) is the number of sorbates in the microscopic unit interface of type τ. Corresponding *A*_ν_^(τ)^ is related, via −*RT* ln *A*_ν_^(τ)^, to the free energy of transferring ν sorbate molecules from the standard state, as shown in our previous paper^16^ (we shall report the values of −*RT* ln *A*_ν_^(τ)^ as the fitting result). In principle, the second term of eq 11 and the second and third terms of eq 12 should all contain the first-order contribution in *a*_2_ to satisfy Henry’s law. At low *a*_2_, however, the first terms of eqs 11 and 12 dominate over the second and third terms of eqs 11 and 12 and the first-order contributions in *a*_2_ in them are in practice not operative. When *a*_2_ becomes large, the higher order contributions in *a*_2_ in the second term of eq 11 and the second and third terms of eq 12 contribute to the adsorption capacity and the first-order contributions are negligible. Thus, unlike the case of eq 6, the *A*_1_*a*_2_ contribution is not needed for the second term in eq 11 and the second and third terms in eq 12. More terms may be added, if necessary, to eq 12 to account for the additional steps in the isotherm.

### Determination of the Parameters

Each cooperative step of our isotherm contains three parameters: *m*, *A*_*m*_^(τ)^, and *N*^(τ)^. For an efficient and unambiguous determination of these parameters, we adopt the following strategy. First, we obtain the following information that can be extracted robustly from the isotherm step under constant *T*: (a) the height of the isotherm step, *h*^(τ)^, (b) the activity at which the isotherm gradient is the steepest, *a*_S_^(τ)^, and (c) the gradient at step b, . See Appendix A for notation.

Using the relationship derived in Appendix A, we solve steps (a)–(c) for *m*, *A*_*m*_^(τ)^, and *N*^(τ)^ by the following procedure: (i) Determine *N*^(τ)^ from steps (a), (b), and (c) via eq A6, i.e., . (ii) Determine *m* from steps (i) and (a) via eq A7, i.e., . (iii) Determine *A*_*m*_^(τ)^ from steps (ii) and (b) via eq A5, i.e., *A*_*m*_^(τ)^ = (*a*_S_^(τ)^)^−*m*^.

**Figure 2:**
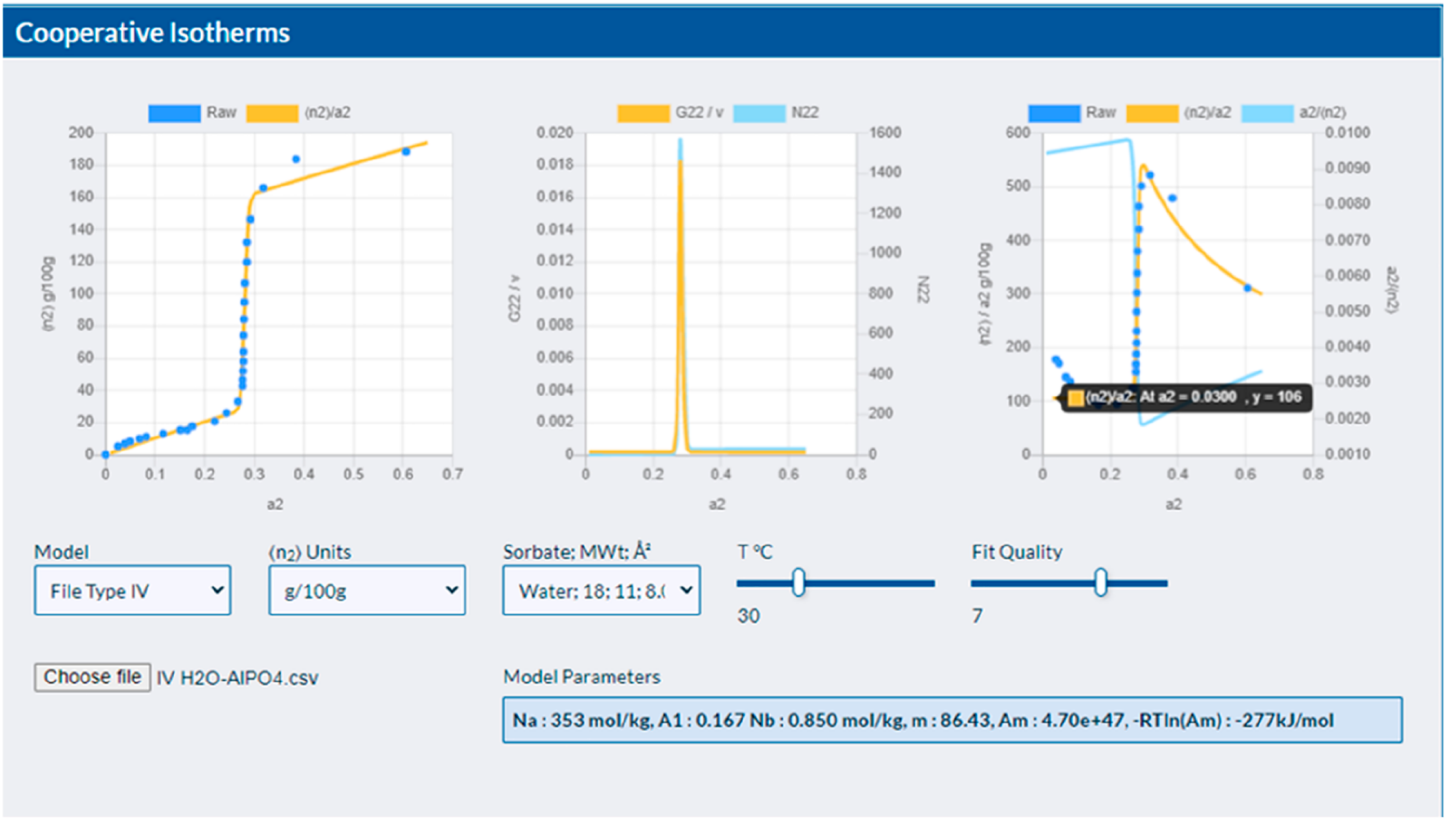
Screenshot of the web-based app that has implemented the theory developed here. The open-source versatile app is freely available at https://www.stevenabbott.co.uk/practical-solubility/Isotherms-Cooperative.php to allow users to test the ideas using their own data sets, loaded into the app as simple .csv files. Additional isotherm data are available therein to demonstrate the wide applicability of our approach. Not only does the interactive app carry out fitting, but it also demonstrates statistical thermodynamics at work in an interactive manner.

How the use of steps (i)–(iii) improves fitting is demonstrated in Appendix B. In practice, experimental uncertainties in the three input parameters can lead to a non-optimal fitting of the entire data set. Hence, the parameters can be used as inputs to a generalized fitting routine, ensuring rapid convergence onto a realistic optimum. Users can readily perform the fitting using, say, the Solver in Excel or a standard routine in MATLAB. The general purpose app has been developed here, which makes it convenient to load the data set of a user and test out multiple fits (Figure 2). It is probable that the different algorithms of an Excel fit and the app fit will give somewhat different fitted values, especially when the experimental data set is sparse around the stepwise increase of an isotherm. Hence, if we take *m* to be a “cluster size”, it at first seems unsatisfactory that the same data set can give different values of *m*. The *m* values are a shorthand for a complex array of molecular arrangements on a surface, and modest errors in experimental data or sample preparation can have significant effects on the fitted values. If the goal is to reveal the behavior of the sorbate around the stepwise increase, then this problem can be overcome via our recent statistical thermodynamic theory, in which the gradient of the ln *a*_2_ – ln⟨*n*_2_⟩ plot gives the overall cluster size precisely provided that the experimental data are measured extensively around the isotherm step.^13−15^ However, if the priority is to fit the overall shape of an isotherm, the fitted value of *m* is a semi-quantitative measure of the sorbate molecules involved; what is important is not so much the precise value of *m* for a given system, but whether it is small, medium, or large. This is a feature of the approach and not a bug.

## Results and Discussion

### Surface Adsorption Followed by Sorbent Structural Changes

#### Hydrogen-Bonded Organic Framework

**Figure 3:**
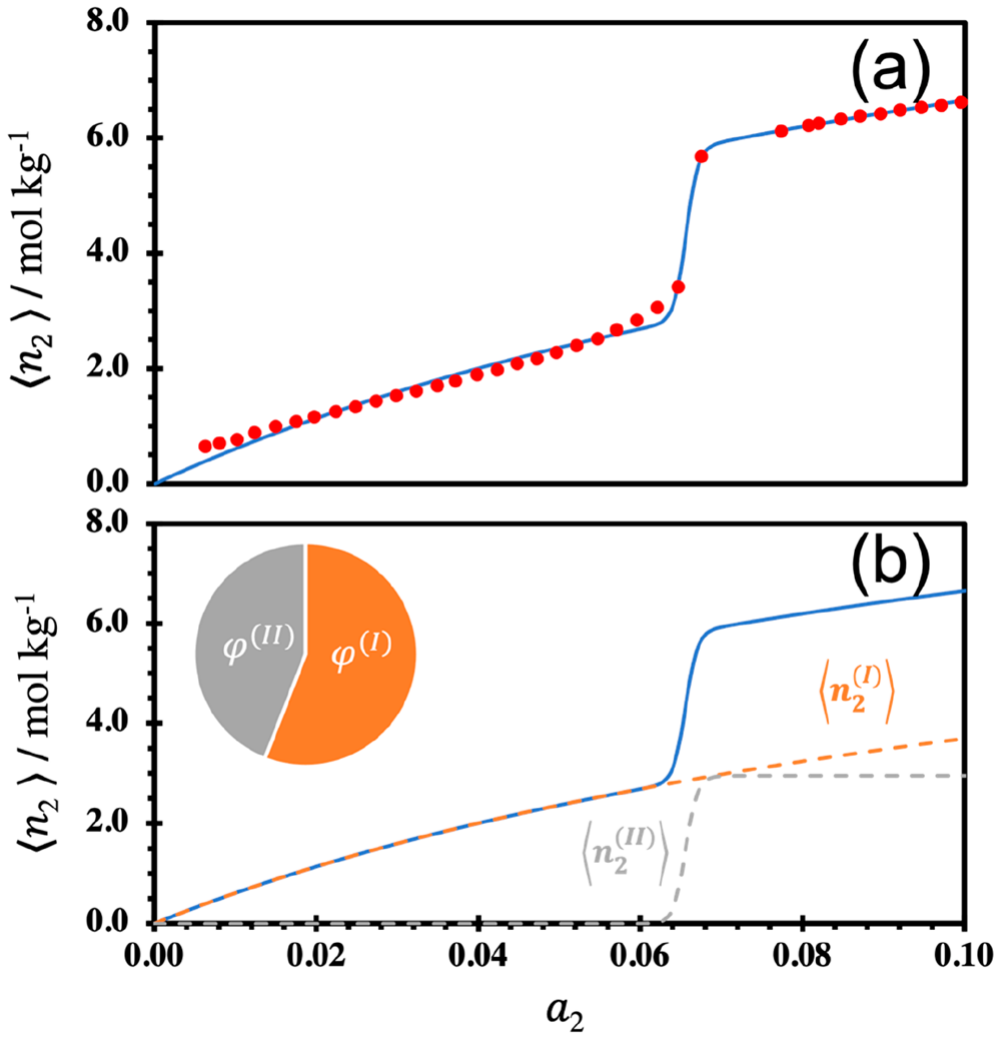
Adsorption isotherm of NH_3_ (g) onto KUF-1a, a HOF, at 298 K,^49^ showing the (a) experimental data points (red circles) on top of the isotherm fit using eq 11 (solid blue line) and (b) isotherm fit using eq 11 (solid blue line) with its breakdown into the simple surface adsorption ⟨*n*_2_^(*I*)^⟩ (dashed orange line) and cooperative adsorption ⟨*n*_2_^(*II*)^⟩ (dashed gray line). The chart in panel b shows the comparative contributions to total sorption from the simple surface (φ^(*I*)^) and cooperative (φ^(*II*)^) sorption components. The fitting parameters are summarized in Table 1.

The heterogeneous isotherm containing the simple surface and cooperative adsorption terms (eq 11) could successfully fit the gaseous adsorption of NH_3_ onto KUF-1a at 298 K (Figure 3a, with the parameters in Table 1), a hydrogen-bonded organic framework (HOF), which was designed specifically to exhibit a type IV isotherm shape to maximize NH_3_ adsorption on the inflection, whose measurement and data have been reported by Kang et al.^49^ The breakdown of this fit into how the contributions from simple surface adsorption ⟨*n*_2_^(*I*)^⟩ and cooperative adsorption ⟨*n*_2_^(*II*)^⟩ change with *a*_2_ will be useful. For which purpose, we define ⟨*n*_2_^(*I*)^⟩ and ⟨*n*_2_^(*II*)^⟩ as
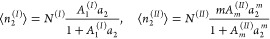
13as the first and second terms of eq 11, respectively. Figure 3b shows that the simple surface adsorption, ⟨*n*_2_^(*I*)^⟩, initially dominates the isotherm up to around *a*_2_ = 0.06, followed by the onset of cooperative adsorption, demonstrated in the inflection. This was attributed to a structural change to the HOF induced by NH_3_ forming hydrogen bonds with the framework, underscored by the lack of inflection for gases incapable of forming hydrogen bonds with the HOF.^49^ This cooperative component represents the influx of NH_3_ molecules into the structure, with around 90 molecules per unit interface (*m*; Table 1) (as emphasized in the Theory and Methods, such numbers should be taken semi-quantitatively). The contributions of each component to the maximum adsorption are also reported in Figure 3b, which shows comparative contributions to total sorption from the simple surface, ϕ^(*I*)^, and cooperative, ϕ^(*II*)^, contributions. Here, ϕ^(*I*)^ and ϕ^(*II*)^ have been defined as ⟨*n*_2_^(*I*)^⟩/⟨*n*_2_⟩ and ⟨*n*_2_^(*II*)^⟩/⟨*n*_2_⟩ at *a*_2_ = 0.1, respectively. For this isotherm, both ϕ^(*I*)^ and ϕ^(*II*)^ are relatively similar, showing a near even distribution of adsorption between the simple surface and cooperative components (Figure 3b).

**Table 1:** Summary of Fitting Parameters Obtained for the Three Datasets Obtained from Eqs 11 and 12

data set	equation	*N*^(*I*)^	–*RT* ln *A*_1_^(*I*)^(kJ mol^–1^)	*N*^(*II*)^	*m*	–*RT* ln *A*_*m*_^(*II*)^(kJ mol^–1^)	*N*^(*III*)^(cm^3^ g^–1^)	*n*	–*RT* ln *A*_*n*_^(*III*)^(kJ mol^–1^)
NH_3_ adsorption onto KUF-1a^49^	11	8.41 mol kg^–1^	–5.10	0.0330 mol kg^–1^	89.6	–605			
water adsorption onto AlPO_4_-5^50^	11	38.9 mol kg^–1^	3.21	1.56 mol kg^–1^	85.2	–273			
CO_2_ adsorption onto PCN-53^32^	12	668 cm^3^ g^–1^	–1.60	21.0 cm^3^ g^–1^	14.1	–15.9	3.25	54.7	–30.9

#### Molecular Sieve

**Figure 4:**
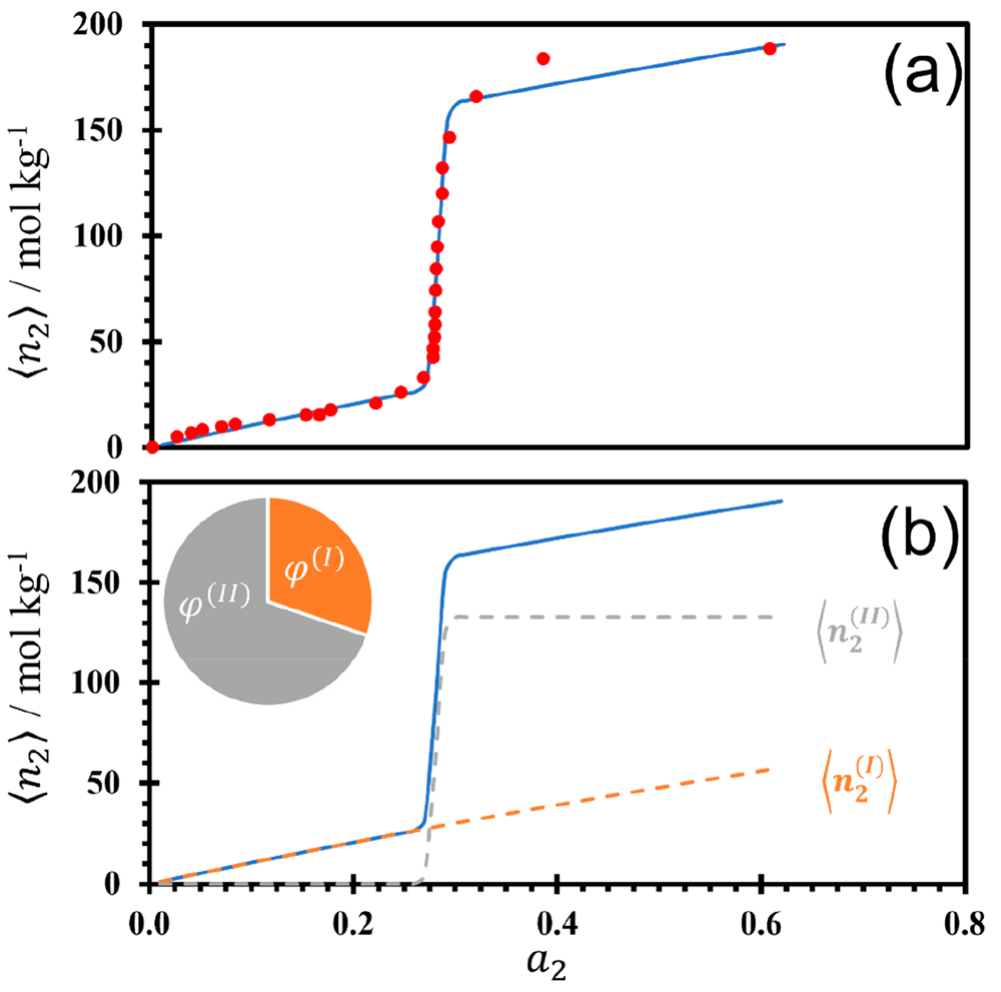
Adsorption isotherm of water onto an aluminophosphate molecular sieve,^50^ AlPO_4_-5 [(AlO_2_)_12_(PO_2_)_12_], at 303 K, showing (a) the experimental data points (red circles) on top of the isotherm fit using eq 11 (solid blue line) and (b) the isotherm fit using eq 11 (solid blue line) with its breakdown into the simple surface adsorption ⟨*n*_2_^(*I*)^⟩ (dashed orange line) and cooperative adsorption ⟨*n*_2_^(*II*)^⟩ (dashed gray line). The chart in panel b shows the comparative contributions to total sorption from the simple surface (φ^(*I*)^) and cooperative (φ^(*II*)^) sorption components. The fitting parameters are summarized in Table 1.

The adsorption of water onto an aluminophosphate molecular sieve, AlPO_4_-5 [(AlO_2_)_12_(PO_2_)_12_], at 303 K, which has a one-dimensional pore channel within its framework structure and has been measured and reported by Tsutsumi et al.,^50^ was modeled by eq 11 (Figure 4a, with the fitting parameters in Table 1). The breakdown into the simple surface and cooperative contributions is shown in Figure 4b. Unlike the previous example of NH_3_ adsorption (Figure 3), Figure 4b shows that, for this isotherm, there was an uneven distribution of adsorption. The contribution from simple surface adsorption is small, at 30% (ϕ^(*I*)^), while the dominant contribution comes from the cooperative adsorption to the pore, with 70% adsorbing to the porous surfaces (ϕ^(*II*)^). The surface of the molecular sieve has few hydrophilic sites^50^ that are occupied at very low water activity with the simple surface isotherm, ⟨*n*_2_^(*I*)^⟩, while the porous channels are not accessed until around *a*_2_ = 0.25 with the cooperative isotherm, ⟨*n*_2_^(*II*)^⟩. Upon fitting, the number of water molecules absorbed cooperatively to each type *II* unit interface can be estimated to be around 85 (*m*; Table 1), potentially indicating a similar size to these porous channels as the HOF discussed above.

#### Sorbent Structural Changes

HOF is among the class of sorbents that can go through structural changes through hydrogen-bond rearrangement.^51,52^ As with the adsorption of NH_3_,^49^ these changes may be likely on sorbate adsorption. Such a structural change of the sorbent, from the perspective of simple physical models, would lead to an increase in fitting parameters. Our rigorous statistical thermodynamic theory,^13−16^ on the contrary, is valid regardless of the structural changes in the sorbent. Note that the gradient of the sorption isotherm, which is determined by the sorbate–sorbate interaction,^13−16^ is also mediated by the interaction with the sorbent, which is incorporated implicitly into our theory.

#### Comparison to Previous Models

Equation 11 was successful in modeling the type IV isotherm. In contrast, our homogeneous cooperative sorption isotherm (eq 6), which has been demonstrated to fit type V isotherms, is insufficient for the type IV isotherm. This is demonstrated by gaseous NH_3_ adsorption onto KUF-1a (Appendix B). Such a shortcoming has been overcome by eq 11. Furthermore, the type IV isotherm of water adsorption to an aluminophosphate molecular sieve was successfully fit using eq 11 (Figure 4), contrasting the previous unsuccessful fit by the model-based approach (i.e., Klotz-derived isotherm).^22^

### Stepwise Isotherms

As discussed in the Introduction, there are two classes of sorption phenomena that exhibit stepwise isotherms: (i) porous materials consisting of different pore sizes and (ii) “layer-by-layer adsorption on a highly uniform nonporous surface”^9^ at a low temperature, which is the strictly defined Type VI isotherm by IUPAC [note that strict layer-by-layer adsorption (ii) involves an infinite number of sorbates and *m*]. Because our focus is on heterogeneous adsorbents, here, we show that our theory, eq 12, can be applied to the Type VI-like stepwise isotherms (i).

#### Porous Materials with Heterogeneous Pore Sizes

**Figure 5:**
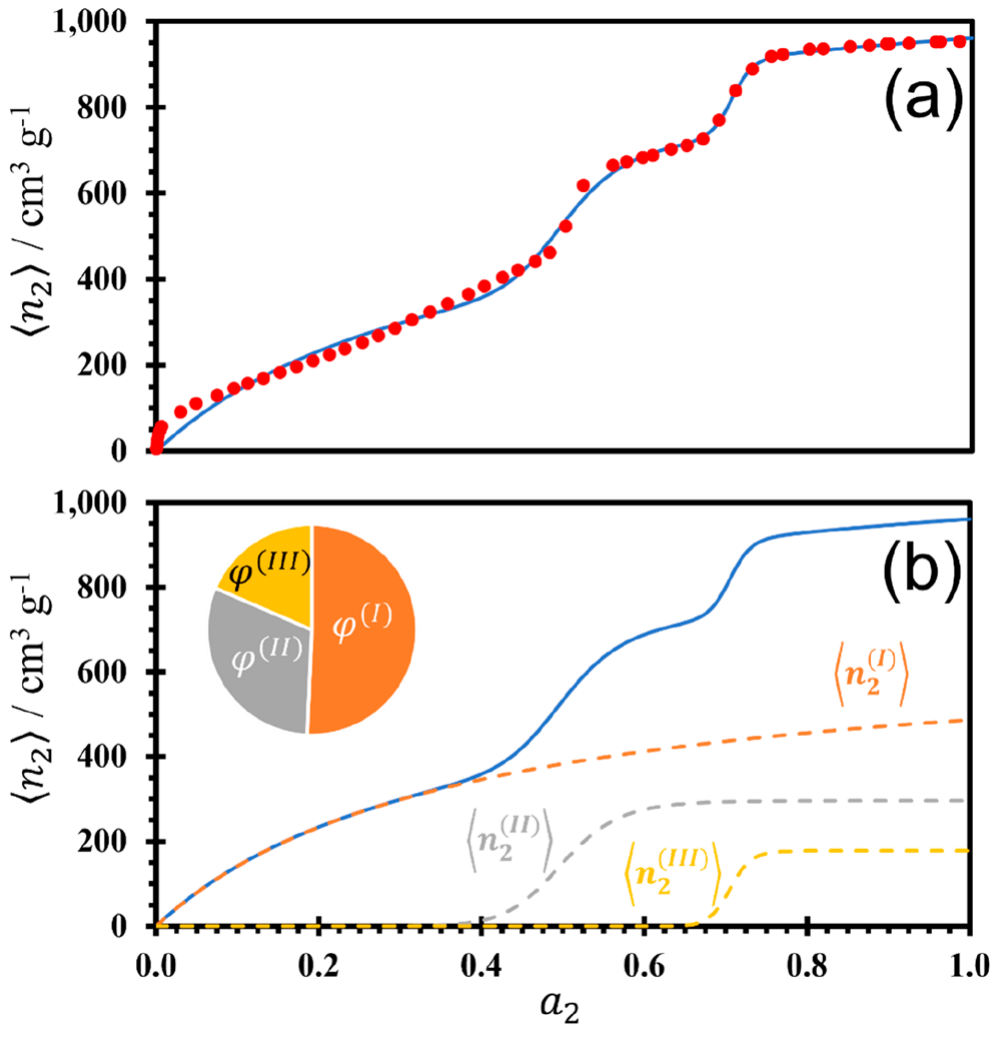
Adsorption isotherm of CO_2_ (g) onto a MOF,^32^ PCN-53, at 195 K, showing (a) the experimental data points (red circles) on top of the isotherm fit using eq 12 (solid blue line) and (b) the isotherm fit using eq 12 (solid blue line) with its breakdown into the simple surface adsorption ⟨*n*_2_^(*I*)^⟩ (dashed orange line) and two cooperative adsorption components, ⟨*n*_2_^(*II*)^⟩ (dashed gray line) and ⟨*n*_2_^(*III*)^⟩ (dashed yellow line). The chart in panel b shows the comparative contributions to total sorption from the simple surface (φ^(*I*)^) and cooperative (φ^(*II*)^ and φ^(*III*)^) sorption components. The fitting parameters are summarized in Table 1.

Here, we apply eq 12 to model the adsorption of CO_2_ onto a metal–organic framework (MOF), PCN-53, at 195 K, which contains micro- and mesopores, measured and reported by Yuan et al.^32^ The adsorption (Figure 5a) can be modeled successfully by eq 12, with the fitting parameters summarized in Table 1. The breakdown into the simple surface adsorption and two cooperative contributions can be found in Figure 5b as well as their respective contributions to the overall sorption. Park and Suh originally attributed to the sequential filling of pores with different sizes.^53^ Even though fitting eq 12 to experimental isotherm data does not yield direct information on the pore size and geometry, the insight into porous sorption can be gained from the mean number of sorbates from the cooperative contributions to eq 12. The number of sorbates, 14 and 55 (*m* and *n* in Table 1, respectively), corresponds semi-quantitatively to the number of sorbates involved in capillary condensation in the pore, and a larger sorbate number is expected when the pore size is large. Subsequently, because a much larger sorbate number is found for the second cooperative component, with 55 for ⟨*n*_2_^(*III*)^⟩, it may be assumed that this adsorption process occurs in a larger pore size to the first cooperative component, with only 14 for ⟨*n*_2_^(*II*)^⟩. This supports the structure of PCN-53, known to contain both micro- and mesopores, suggesting that the larger mesopores are filled after the smaller micropores.

#### Comparison to the Previous Models

The strength of our approach, in contrast to the previous model-based approaches, is 3-fold: (i) the minimum number of postulates involved in the theory, (ii) the parameters (cluster number and sorbate transfer free energy) with a clear statistical thermodynamic interpretation that leads directly to mechanistic insights, and (iii) the versatile nature in which multiple stepwise (heterogeneous cooperative) isotherm formulas can be constructed in a consistent manner as the single-step (homogeneous cooperative) isotherms. Strength (i) contrasts with the need for a large array of fitting parameters required for each region of an isotherm plot.^34^ Strengths (ii) and (iii) contrast with the need for different forms of fitting equations for each experimental data set.^35^

#### Cooperativity in Porous and Planar Surfaces

Thus, we have shown that eq 12 can be applied to heterogeneous porous materials containing different pore sizes. Equation 12 can identify the number of sorbates adsorbing cooperatively together, which follows the initial surface adsorption. Such a versatile nature comes from the minimum number of assumptions involved in our approach. As we emphasized in the Theory and Methods, the fitted value of *m* depends upon the fitting quality as well as how dense/sparse the isotherm data are around the stepwise increase; hence, *m* should be taken as a semi-quantitative measure of the sorbate cluster number. A more precise evaluation of the overall sorbate cluster number can be achieved with statistical thermodynamics via a ln–ln plot of an isotherm.^13−15^ The numbers of sorbates involved would be useful in linking the molecular distribution of sorbates at the interface from simulation to the macroscopically measured isotherm. Nevertheless, we have demonstrated that the use of our approach in conjunction with the experimental insights on the sorption mechanism can lead to the quantification of the cooperative behavior of the sorbates that take place at the interface.

## Conclusion

Developing models have been central to the study of sorption isotherms. However, the co-existence of more than 80 different models and the reports of multiple models with different parameters being able to fit an isotherm equally well^54^ have raised doubts over whether fitting models to an experimental isotherm can really aid the understanding of the sorption mechanism.^29^ This has motivated us recently to develop a universal statistical thermodynamic theory of sorption, linking the functional shape of an isotherm to sorbate–sorbent and sorbate–sorbate interactions.^14,15^ On the basis of this foundation, we have developed a 2-fold approach to model isotherms, via the expansion of sorbate–sorbate interactions^14^ and the cooperative sorption theory.^16^

Our aim is to model a wide variety of isotherms from a simple and systematic approach. Here, we have extended the cooperative sorption theory^16^ to heterogeneous surfaces, composed of either different pore types or pores along with simple surfaces. Postulating the existence of the microscopic interfacial units of different types has led straightforwardly to the superposition scheme of isotherms, each responsible for a simple surface and several different pore types. Our heterogeneous cooperative sorption theory could successfully fit Type IV isotherms (increase at low activity followed by a sigmoidal increase) and stepwise isotherms of porous materials containing micro- and mesopores.

The strength of our theory lies in its generality, which does not make any assumptions on the shape and geometry of the interface, except for the finite-ranged nature of the interface and the existence of independent microscopic interfacial subsystems.^16^ This is why our approach is applicable to complex cases involving multiple pore sizes and sorbate-induced structural changes, leading to clear mechanistic insights without the need for *a priori* assumptions on the sorption mechanism.

Instead of introducing explicit mechanistic assumptions on pore size dependence of capillary condensations, as customary in constructing sorption models, in our theory, the number of sorbates sorbing cooperatively can be determined directly from an isotherm, which provides mechanistic insights. Instead of assuming the mechanism of adsorption-induced sorbate structural changes as customary, in our theory, such an effect is captured quantitatively via the cooperative number as well as the free energy required to bring the sorbates together. When the sorption mechanism has been established unambiguously, a model-based approach can be useful. However, when there is no prior knowledge of the sorption mechanism, our approach is advantageous. Our approach can be applied directly to the isotherm itself in a robust manner, leading automatically to mechanistic insights into sorption. Such generality of our approach is complementary to the traditional model-based approaches based on *a priori* assumptions on the sorption mechanism. Indeed, our statistical thermodynamic strategies^14,16^ have been demonstrated to be successful in modeling a wide variety of isotherms through the parameters with physical meaning.

## Figures and Tables

**Figure 6:**
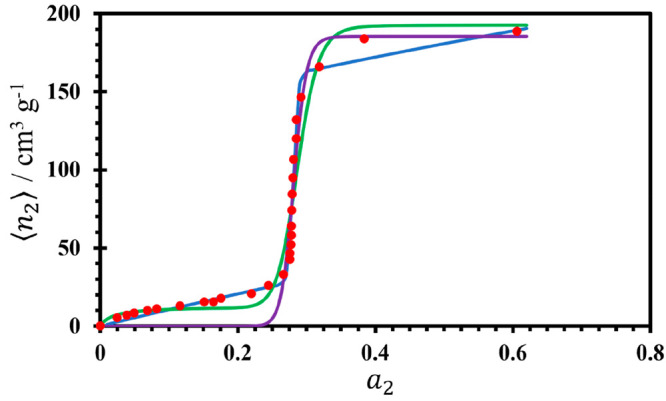
Comparison of different isotherm models on fitting the adsorption isotherm of water onto an aluminophosphate molecular sieve,^50^ AlPO_4_-5 (Figure 4), showing the experimental data points (red circles) on top of the isotherm fit using eq 11 with (solid blue line) and without (solid green line) the height, position, and maximum gradient of the cooperative step incorporated, as well as the isotherm fit using eq 6 for Type V isotherms (solid purple line).

## Appendix A

Here, we focus on one of the cooperative terms in eqs 11 and 12 and show how the parameters can be determined robustly. Here, we focus on the τth term

A1

and calculate its first- and second-order derivatives under constant *T* as

A2

and

A3

Under constant *T*, *a*_2_ is the only variable, hence the notation for single-variable differentiation has been adopted. Here, we define the point *a*_2_ = *a*_S_^(τ)^ at which the gradient of the isotherm is the steepest, which can be evaluated by solving , leading to

A4

and

A5

when *m* is sufficiently large. The isotherm gradient at *a*_2_ = *a*_S_ can be evaluated by substituting eq A5 into eq A2 as

A6

In addition, the height of the isotherm step, *h*^(α)^ (i.e., the difference in the amount of sorption before and after saturation), can be evaluated from eq A1 as

A7

Thus, the three parameters for the *τ*th term of the isotherm, *A*_*m*_^(τ)^, *N*^(τ)^, and *m*, can be evaluated from the easily obtainable features of the isotherm, *a*_S_^(τ)^, , and *h*^(τ)^, by solving eqs A5–A7.

## Appendix B

Here, we demonstrate that (i) isotherm fitting can be improved using the information on height, steepest gradient, and activity at the steepest gradient (Appendix A) and that (ii) the homogeneous cooperative isotherm (eq 6) is insufficient to capture the strong increase of the isotherm at a low activity. This is demonstrated in Figure 6 using the adsorption isotherm of water onto an aluminophosphate molecular sieve,^50^ (AlO_2_)_12_(PO_2_)_12_. Using the information on height, steepest gradient, and activity at the steepest gradient improves fitting, especially around the region where the isotherm increases most steeply (Figure 6, blue line). Since non-liear fitting is not a straightforward procedure, an unsatisfactory fit (Figure 6, green line) may be obtained when executed without such additional information (i). Moreover, the Type V isotherm model (eq 6) cannot capture the initial rise of the isotherm (ii).
